# Threats to and Opportunities for Low-Income Homeownership, Housing Stability, and Health: Protocol for the Detroit 2017 Make-It-Home Evaluation Study

**DOI:** 10.3390/ijerph182111230

**Published:** 2021-10-26

**Authors:** Roshanak Mehdipanah, Margaret Dewar, Alexa Eisenberg

**Affiliations:** 1 Department of Health Behavior and Health Education, School of Public Health, University of Michigan, Ann Arbor, MI 48109, USA; alexae@umich.edu; 2 Urban and Regional Planning Program, Taubman College of Architecture and Urban Planning, University of Michigan, Ann Arbor, MI 48109, USA; medewar@umich.edu

**Keywords:** housing instability, health inequities, Detroit

## Abstract

Few studies have examined the combined effects of affordability, housing conditions and neighborhood characteristics on the housing stability and health of low-income homeowners. We begin to address these gaps through a mixed-method study design that evaluates the Make-it-Home program (MiH) in Detroit, Michigan, aimed at helping low-income tenants become homeowners when their landlords lose their homes to tax foreclosure. We compare the ‘intervened group’ of MiH homeowners to a ‘comparison’ group of similarly situated households whose homes experience property tax foreclosure at the same time. The comparison group represents the likely outcomes for the participants had they not participated the program. Participants will be surveyed twice (intervened group), or once (comparison group) per year over a three-year period, regarding their housing and neighborhood conditions, health, life events, and socio-economic status, including income and employment. We will use property and neighborhood census data to further examine the conditions experienced. The findings for policy and program development from this study are timely as the nation faces a chronic shortage of affordable housing for both purchasers and renters. The results suggest ways to improve the MiH program and lay out approaches for researchers to navigate some of the complexities associated with this type of research.

## 1. Introduction

A growing body of public health research has considered the health effects of homeownership. Compared to renters, owners tend to demonstrate lower mortality and morbidity rates [1,2,3]. Although household income and education account for some of this association, homeownership is hypothesized to improve health and socioeconomic opportunities through wealth creation, residential stability, better housing conditions and neighborhood environments, and a greater sense of control and security [4]. Yet, scholars have not thoroughly examined the health effects of homeownership across different subpopulations and geographical contexts [5], raising questions regarding for whom and under what conditions homeownership can benefit health.

Variation in the access to homeownership has important implications for health equity, as the historic and contemporary discrimination in US housing and labor markets make Black and Latinx populations less able to attain and sustain homeownership, and therefore less likely to realize its associated health benefits [6]. Lower incomes and racial segregation lead home buyers in these groups to purchase housing in neighborhoods with older, poorer quality housing stock, fewer neighborhood amenities, and lower home values that appreciate more slowly over time. Lower home values reduce the positive association between homeownership and mortality at the neighborhood level, suggesting that the health benefits of homeownership are contingent upon neighborhood conditions that also negatively affect home values [7]. The fluctuations in house prices put any progress in housing equity at risk [8,9,10]. The last financial crisis highlighted the unequal risks of homeownership when mortgage brokers systematically targeted households in neighborhoods dominated by people of color for subprime and predatory loans, resulting in disproportionate rates of default and repossession in these neighborhoods [11,12]. Research has linked neighborhood mortgage foreclosure rates to various health outcomes including hospital visits, poorer self-rated health and high systolic blood pressure [13,14,15].

Persistent racial inequities in wealth and health mean that low-income Black and Latinx homeowners face a greater risk of housing loss due to unexpected expenses or income losses [16]. Sustaining homeownership is key to a households realizing of the benefits of homeownership [17]. Among a sample of low-income buyers participating in an affordable, nationwide homeownership pilot program, one-third experienced a major home repair cost that they could not afford [18]. The substantial disrepair of a home can leave homeowners with few choices other than to move [19]. Several studies associated a poor and worsening health status with a higher risk of default and foreclosure [20,21] due to high medical bills and employment loss that could cause income declines and the loss of health insurance [22,23]. Studies also showed that unexpected life events such as job loss, death in the family or divorce could result in income loss or costly bills that could lead to default on housing-related payments [17,18].

These findings raise questions about whether homeownership is indeed beneficial for all people. Depending on the circumstances, homeownership may already cause low-income households to take on greater financial risks, and these may further increase their risk of unstable housing and poor health [24]. Most of the public health literature to date has focused on mortgage costs and few studies have considered how costs, such as property taxes, repairs and utilities, can affect the health and housing stability of homeowners among low-income households of color [25,26]. Previous studies have not sufficiently examined the combined effects of affordability, housing conditions and neighborhood characteristics on the housing stability and health of low-income homeowners. 

### Research Purpose

We begin to fill these gaps in the literature by examining the combined threats of poor housing conditions, unaffordable housing costs (taxes, home insurance, utilities), and neighborhood disinvestment, together with socioeconomic characteristics, life events, and health, on the housing stability and subsequent health of low-income homeowners in Detroit, Michigan. Therefore, we consider the bidirectional pathways between housing stability and health outcomes [23,27,28]. Our prospective study design enables us to examine not only what undermines housing stability for new owners but also to examine whether homeownership is still beneficial, and under which circumstances, over time. To conduct this study, we evaluated the Make-it-Home program (MiH), aimed at helping low-income tenants become homeowners when their landlords lose their homes to tax foreclosure. We compared the “intervened” group of recent MiH homeowners to a “comparison” group of similarly situated households whose homes went into property tax foreclosure at the same time. While tax foreclosure threatened the housing stability of both groups, the intervention provided some households with a pathway to ownership. The comparison group represents what likely would have happened to the participants in the absence of the program. In part because of the rush to implement the MiH program to prevent properties from entering the tax auction, many of the comparison group members were similar to MiH households but did not respond quickly enough to phone calls or property visits to meet the deadline for enrolling participants in the MiH program. 

## 2. Materials and Methods

### 2.1. Study Setting

Detroit, Michigan is a compelling setting for this research due to its historical and contemporary housing context and socioeconomic and physical challenges. The majority of the population of Detroit are Black (78% in 2019), and more than one third (35%) of all residents lived in poverty in 2019 [29]. The demand for Detroit’s housing is weak owing to a legacy of segregation and racial discrimination, population loss, and the concentration of low-income households. The housing stock has suffered a considerable disinvestment, and the city lost 45,000 (12%) of its housing units between 1990 and 2013 [29]. The housing is also aged (nearly 80% of housing units were built before 1960) and of a low-value (the median housing value was USD 49,200 in 2019); and thousands of units, especially those affordable to low-income households, are in inadequate condition [30]. Twenty-seven percent of Detroit’s housing units were vacant in 2017 [31]. The city experienced large numbers of mortgage and property tax foreclosures during and after the last recession [32]. Between 2005 and 2014, nearly 30% of Detroit’s mortgageable properties underwent foreclosure [33]. Declining home values, inflated property tax assessments, a rising poverty rate, and growing fiscal distress in the city contributed to a subsequent wave of tax foreclosures due to unpaid property taxes. Between 2010 and 2017, more than 47,000 properties with occupied structures in Detroit entered the tax foreclosure auction (data obtained from the Wayne County Treasurer, 2017, through a Freedom of Information Act request by J. Paffendorf, CEO, Loveland Technologies). Although Detroit was once renowned for its high level of Black homeownership, the city’s homeownership rate decreased from 54.9% to 47.2% between 2000 and 2018, and Black homeownership decreased from 53% in 2000 to 47% in 2019 [30,34,35,36].

### 2.2. Make It Home Program

In response to the large numbers of households losing their homes to tax foreclosure, the Make it Home (MiH) program was created in 2017 through a partnership between Quicken Loans Community Fund (QLCF—a foundation focused on community development), the United Community Housing Coalition (UCHC—a non-profit organization focused on preventing evictions, foreclosures and homelessness among Wayne County’s low-income residents), the Wayne County Treasurer and the City of Detroit government. State law permits a city, village, or township to purchase tax foreclosed properties within its jurisdiction prior to tax auction through the “Right of First Refusal” by paying the minimum bid of delinquent taxes, fees, and interest [34]. In its first year of implementation, city officials used the Right of First Refusal to help 80 Detroit renters to purchase their homes, which had entered tax foreclosure through no fault of the renters. In August 2017, the City of Detroit used donated funds from the QLCF and participants’ savings (approximately USD 500 each) to purchase participants’ tax-foreclosed homes from the Wayne County Treasurer and transferred them to the UCHC, who then sold them to their occupants. The homes ranged in price from USD 2000 to almost USD 5600, and the UCHC provided a zero percent interest *land contract* for each resident who was not able to immediately pay the cash price for the home. The land contracts meant that owners could pay the balance of the cost of the home over time; land contracts were agreements between the UCHC and the purchasers that did not involve a bank. Banks and mortgage brokers rarely write mortgages for the small sums involved in housing purchases and most of the purchasers would most likely not have been deemed creditworthy by the bank lenders. The homes were located throughout the city. 

The program expanded annually through 2019, and approximately 1100 households participated (a moratorium on tax foreclosures in 2020 and 2021 meant that no new households enrolled those years). Through additional funding from the QLCF and other sources in 2018, the UCHC established a program to provide aid to homeowners in the form of small grants or loans to address repair needs. 

According to the UCHC and QLCF leaders, the goals of the program were, first, to prevent housing loss by enabling tenants to remain in the homes that landlords were losing to tax foreclosure. If the houses ended up at the tax auction, investors would be the most likely purchasers causing potential increases in rents and evictions [32]. In addition, the program aimed to make longer term homeownership possible and to enable purchasers to gain equity. The hope was that the program would provide individuals with opportunities to become and remain homeowners, preserve properties, and reinforce neighborhood housing markets. However, past research suggests that several factors, including, for instance, poor housing conditions, high housing cost burdens (including property taxes, home insurance, utilities), and low neighborhood quality, may undermine these program goals [11,16,18,25]. Little evidence exists on the effectiveness of such a program for improving long-term housing stability and health by preventing displacement and encouraging homeownership.

Therefore, this study aims to examine the impact of the program on preserving housing stability and on improving health among a group of low-income tenants who transition to homeownership, in comparison to a group of similar households. In doing so, we expect to gain insight into the health and housing stability impacts of tax foreclosure and on the factors that threaten or reinforce housing stability for low-income homeowners.

#### Study Design and Sample

This three-year study compares a group of 2017 MiH participants’ experiences to the experiences of a group of UCHC client households, whose homes also went through property tax foreclosure in 2017 but who were not able to participate in the MiH program. The UCHC recruited MiH participants from the list of the organization’s clients who were renters. At the time of foreclosure, the comparison group consisted of the UCHC clients who were prepared to purchase property at the auction: tenants (who likely were eligible for MiH) (35%), land contract holders (4%), the family members of those who had owned the houses (50%), and individuals who had no legal connection to the house they occupied or had other arrangements (11%). Similar to MiH participants, the comparison group members tried to purchase houses following tax foreclosure but did so by participating in the tax auction (the UCHC bid for properties on behalf of the comparison group members). The previous tenants in the comparison group were in the same position as MiH participants at the start of the MiH program; those who became owners of houses were in a similar position after the program began. The houses in the MiH program and the comparison group had a similar period of tax delinquency and may have been in similar physical conditions. Therefore, the comparison group provided an insight into what MiH participants might have experienced in the absence of program intervention. Because the MiH participants and the comparison group members all sought to become homeowners, we avoided the problem of self-selection bias.

**Figure 1:**
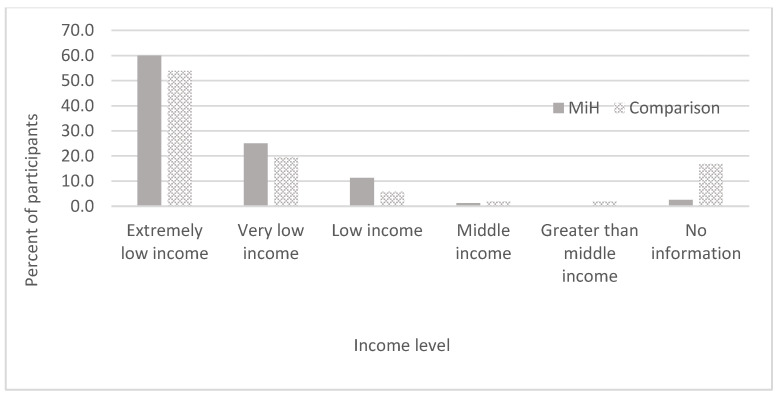
Percent of MiH and comparison participants by level of self-reported income in 2017. Note: Extremely low income = less than or equal to 30% of area median income (AMI) for 4-person households; Very low income = greater than 30% and less than or equal to 50% of AMI; Low income = greater than 50% and less than or equal to 80% of AMI; Middle income = greater than 80% and less than or equal to 100% of AMI; Greater than middle income = greater than 100% AMI [37,38].

According to the information that the UCHC collected at the time of recruitment into either MiH or the effort to purchase houses at auction, most households in the MiH program and the comparison group had incomes at or below 30% of area median income, defined by the U.S. Department of Housing and Urban Development as “extremely low income” (Figure 1). Other research on low-income homeowners has not focused on households with incomes of this level. Therefore, this research is unusual in that it explores the limits and potential benefits from homeownership for extremely low-income households who are often precariously housed as renters and who have limited access to homeownership and conventional mortgage financing [35,36].

The study is a formative evaluation in collaboration with the UCHC. As part of the collaboration, the UCHC reviews drafts of interim reports and survey questionnaires. So far, the researchers have met with the UCHC at least once per year to share findings and discuss implications. As a result of this process, the QLCF and others funded a repair program for MiH and the comparison group participants. The UCHC also distributed information on funding for repairs and on ways to avoid property tax foreclosure. 

The MiH participants are interviewed every six months while the comparison group members are interviewed yearly. The additional semiannual interviews with the MiH group focus on program experiences, health, life events, and perceptions about housing and neighborhood conditions while also serving as an opportunity to try to improve retention for future interviews. The additional interviews also provide an opportunity to monitor potential threats and changes to housing stability and health. Baseline interviews were completed over a year, resulting in the staggering of subsequent surveys. All interviews will be completed by late 2021.

In addition, we are collecting data from publicly available sources about the properties that MiH participants and comparison group members purchased. Because the MiH participants totaled 80 households, and the comparison group, though larger (154 households), had benefited less from their work with the UCHC, we expected a difficulty in conducting enough interviews to make generalizations about each group and to understand the differences between them. Therefore, we hope to use property data to obtain indicators of the housing situation each group experiences and to help place the interview samples in the context of all MiH participants and all comparison households. 

### 2.3. Recruitment

For both the MiH and comparison groups, the study participants had to be 18 years or older at the time of the baseline interview and were either part of the 2017 MiH program or were other UCHC clients who had tried to purchase their houses at the 2017 tax auction. The UCHC provided contact information for the participants and informed them that researchers would telephone them. The UCHC won the bid on behalf of 22 percent of the comparison group members; others needed to find alternative ways to address their housing following tax foreclosure, resulting in different experiences we expected to capture through the surveys discussed below. The recruitment of participants was completed in 2019. Upon the completion of each interview, the interviewee received a USD 25 gift card. Participants who complete all the interviews will receive an additional USD 100. 

### 2.4. Surveys

We designed questionnaires to capture the characteristics of individuals, households, properties, and neighborhoods that might affect housing stability and health. Survey questions drew from several prior surveys that considered housing, neighborhood and health characteristics [2,39,40,41,42,43]. The questionnaire included closed-ended response questions and a section (E) that consisted of a small number of open-ended questions. 

Section A of the surveys provided information on the housing tenure of the participant (owner, renter, or other housing arrangement) and whether they resided in the home they purchased through the MiH program or the auction. We used responses from these questions to determine the subsequent questions that the participant was asked.

The baseline interviews, included in Supplement File S1, asked questions about issues that may affect housing stability: previous homeownership experience, opinions about the condition of and satisfaction with the house and the neighborhood, household composition, marital status, employment, income, health, and housing costs. In addition, we asked questions about life events such as divorce/separation or the death of a loved one that could have financial and health impacts, placing households at greater risk of housing instability. Open-ended questions were asked regarding the experience of working to purchase a house with the UCHC. Baseline surveys lasted approximately one hour.

Follow-up surveys for 6 months (MiH), 1-year (MiH and comparison), and 1.5 year (MiH) intervals consisted of fewer questions focusing on changes in household composition, financial situation, employment, health status, and perceptions of housing conditions and the neighborhood environment. Based on the responses to section A given at baseline and a screening question asking about their living arrangements, we asked participants additional questions related to homeownership, renter status, or another housing arrangement. Participants completed questions based on whether they continued to live in the MiH house or had temporarily or permanently moved out of the house. For the comparison group, participants answered questions about whether they were temporarily or permanently out of the house they tried to buy at the auction or were still living in the house that had been foreclosed. The follow-up surveys consisted of approximately 25 questions and lasted about 30 min. 

Detroit experienced a major surge in COVID-19 cases and deaths from COVID-19 in spring 2020. In the follow-up interviews, we modified the questionnaires to capture some of the health, economic, and social effects of COVID-19 in the life events section. For questions related to job loss, financial difficulties, and serious illness, we asked participants follow-up questions about whether this occurrence was related to the pandemic. 

We conducted baseline interviews with 49 MiH participants and 39 comparison group members. We completed 1-year interviews with 38 MiH and 25 comparison group members who had completed baseline interviews. Due to the difficulty of reaching participants by phone and the resulting delays, in October 2020, we decided to proceed with the final interviews with participants who had completed a baseline interview by October 2018. The aim is to complete the data collection and final interviews, within one year of each other, by late fall 2021. The goal is for the interviews to reflect the status of participants at about the same time since their program participation in 2017.

The final questionnaire is comparable in length to the baseline with similar questions permitting longitudinal comparisons. Specific questions will depend on whether the interviewee is a homeowner, a renter, or in another housing arrangement and whether the interviewee remains in the house purchased through the program or the house that was part of the 2017 tax auction. This questionnaire will consist of approximately 50 questions and will take about one hour.

### 2.5. Additional Measures of House Condition and Neighborhood Environment

Although we asked participants about their housing and neighborhood conditions in the interviews (sections B and C), we will supplement the interviewees’ opinions with measures of property and neighborhood conditions (mentioned below) to see if a ranking of opinions matches the rankings of these additional measures. If the measures correspond in at least 75 percent of cases, we will use the property and neighborhood measures to assess the housing conditions and neighborhood environments experienced by those who were not interviewed.

Using an instrument developed and tested with community development leaders in Detroit prior to this project, we will analyze the exterior condition of MiH houses and houses that the UCHC tried to purchase at the 2017 auction. We will use photos from Google StreetView for 2013 and 2018 to judge the conditions several years before the 2017 foreclosures and the condition immediately after purchase by MiH participants or the UCHC’s effort to purchase at the auction. We will look at changes in condition between the two years to assess disinvestment over that period. The exterior condition assessments can show major structural problems such as a collapsing roof or a crumbling foundation and reveal neglect in the disrepair of steps and the need for painting, for instance. Although these indicate general repair needs, they cannot identify important interior conditions such as a missing furnace or lack of plumbing.

For measures of neighborhood condition, we will use two indicators that interviewees mentioned frequently in baseline interviews when asked about their neighborhoods: crime and the prevalence of vacant structures and vacant lots. First, we will count incidents of violent crime within one quarter mile of each house using crime data available through the Detroit open data portal for a six-month period about one year after the first MiH participants purchased their houses. We will compare the incidence of crime near the MiH houses with that near the comparison participants’ houses. We will also compare these conditions to a random sample of other houses across the city to analyze whether purchasers live in houses with violent crime rates that differ from the experiences of other city residents. 

We will count vacant lots and boarded or open/vacant houses as a percentage of all properties on each block face where an MiH or a comparison group member bought a house; open and vacant houses indicate continued neighborhood disinvestment. Vacant lots result from the demolition of derelict structures. We will calculate the percentage of vacant lots that are owned by adjacent owners, the MiH or the comparison purchaser, and the Detroit Land Bank Authority (DLBA). The purchases made by nearby owners are shown to have positive effects on neighborhoods [44,45]. The MiH or comparison purchasers’ ownership of adjacent lots may indicate a commitment to stay in the house. The DLBA holds land that does not sell in two tax auctions following tax foreclosure; this ownership indicates very weak market conditions and continuing disinvestment.

### 2.6. Property Data

Because all MiH and comparison participants could not be reached for interviews, and our response rate declined with the subsequent interview waves, we have been collecting additional information on the 80 properties, the purchasers that UCHC had originally identified, the 154 comparison group’s houses offered at the tax auction, and the UCHC clients who tried to purchase at the auction to understand some of what happens to the houses and the people involved. This additional information can provide insights on housing instability by revealing indicators of housing loss or further housing investment. These data are publicly available but are not integrated so must be collected from numerous sources to create a dataset that we can analyze. We expect to learn which houses the new MiH and comparison owners have subsequently sold and at which prices, the owners’ subsequent mortgages, new owners’ purchases of adjacent lots, levels of property tax delinquency, and the participation in various programs to prevent tax foreclosure, properties’ probate status when an owner has died, and house vacancy. The assessor’s data indicate the taxpayer of record, tax exemption for owner occupancy, and recent arm’s length sales; the county treasurer’s website has information on tax delinquency and participation in payment plans to prevent tax foreclosure; a private property data vendor provides U.S. Postal Service data on vacancy updated every two months; the 36th District Court has records of tenant evictions; and the county register of deeds has records that include property sales and transfers, judgments of tax foreclosure, mortgages, mortgage foreclosures, probate processes, and some land contracts. 

### 2.7. Data Analysis

We will examine the impact of the MiH program on participants’ housing stability and, consequently, its impact on health over time compared to the experience of those who did not participate in the program. We will also compare the prevalence of characteristics that increase risk of housing instability among the MiH participants compared to others. Since we anticipate a small sample, we will use histograms, Chi-square (to test the independence of MiH and comparison samples) and *t*-tests (to test for differences between the means of variables for the MiH and comparison samples) to compare the distribution of each of the indicators expected to influence housing stability and health outcomes over time. 

### 2.8. Ethics, Funding and Dissemination

The University of Michigan Institutional Review Board (HUM00142978) approved the methods of recruitment, the process of obtaining participants’ informed consent, the questionnaires, and the compensation in April 2018. The funding for this evaluation was received from QLCF and from Poverty Solutions at the University of Michigan. The final findings will be disseminated through program reports with recommendations, peer-reviewed journals, and national conference presentations.

## 3. Baseline Results

### Characteristics of the MiH and Comparison Group at Baseline

At baseline, 74 of the 80 originally identified MiH purchasers continued to live in the houses they had purchased or were working towards purchasing. The UCHC sold six of the remaining properties to the other individuals, and four properties were not yet sold through a deed or land contract. The sales to individuals not originally in the program were primarily situations where the original MiH participant chose to leave the program. In such cases, the UCHC identified potential buyers who would ideally occupy the home. The UCHC was successful in purchasing 34 of the 154 comparison group members’ properties at the tax auction (all but 2 of the 34 properties had been sold to the originally identified comparison purchasers). The remaining 121 comparison group households (120 whose houses the UCHC did not purchase and one whose house the UCHC sold to someone else) faced unknown housing situations. 

**Figure 2:**
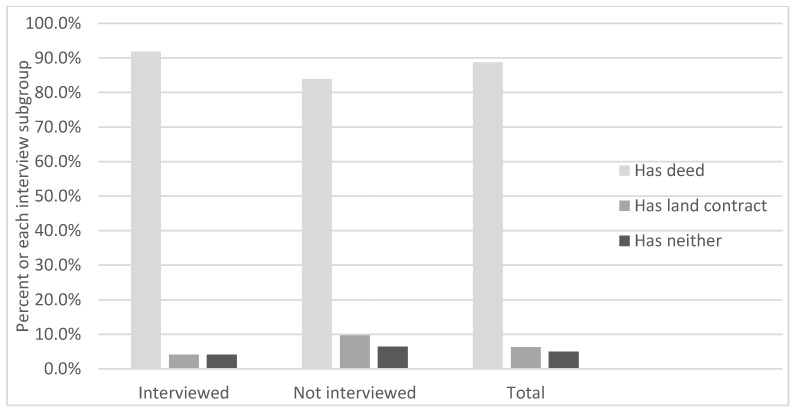
Percent of each Make-it-Home interview subgroup by ownership status.

The 49 MiH interviewees were those we could reach via many phone calls attempted over more than a year; they were not a random sample. Therefore, we were concerned that their situations would not necessarily be representative of all 80 of the participants. As Figure 2 shows, those who had land contracts or had neither a land contract nor a deed, were slightly underrepresented in the interviews, and those who received deeds were slightly overrepresented.

**Figure 3:**
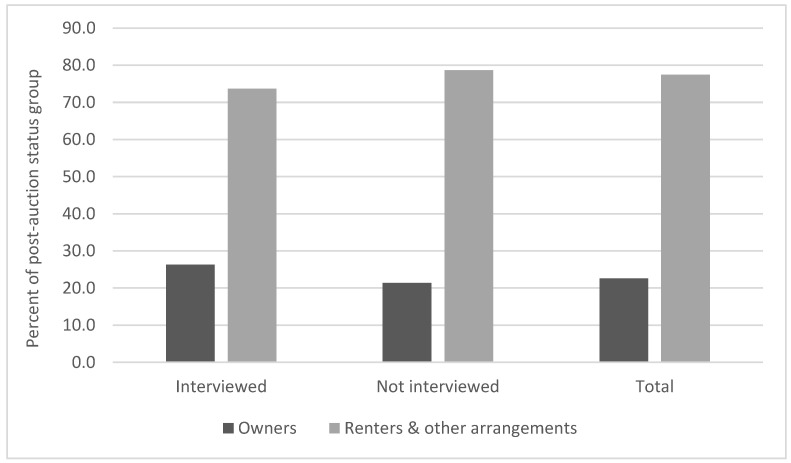
Percent of comparison interview subgroup by post-auction status.

The comparison group respondents were also not a random sample of the households on our list; they were those whom interviewers were able to reach and interview during many phone calls on different days and at different times over more than a year. As Figure 3 shows, the 39 interviews slightly overrepresented those who were owners after the auction and somewhat underrepresented those who were renters or had other arrangements.

Table 1 presents baseline characteristics of the MiH and comparison interviewees. Overall, the analysis of the characteristics showed that the baseline and comparison interviewees were similar at the start of their homeownership or their loss at the auction, as we had hypothesized they would be. The divergence of characteristics or the continued similarity over time could, therefore, show whether the MiH program will lead to more housing stability and improvements in health.

Using Chi-square tests and *t*-tests to examine the relationships between frequencies or means, respectively, we saw no significant differences in age, gender, race, or employment status. 

Because the MiH program resulted in ownership, at baseline, forty-seven participants were classified as owners with deeds and two participants were classified as owners in process. However, for the analysis, both groups were aggregated as owners. The comparison group included some owners (33%), some renters (38%), and those who had other arrangements (28%). All MiH interviewees continued living in the homes that had been tax foreclosed, while only 46% of the auction participants continued living in those homes. 

Two indicators that we hypothesize will help determine future housing stability are housing condition satisfaction and neighborhood satisfaction. The groups differed significantly (*p* < 0.05) in housing satisfaction with the MiH participants more likely to express satisfaction than all of the comparison group interviewees. However, when considering only the owners within the comparison group, no statistically significant differences existed between the two groups (*p* > 0.05—results not shown). No significant differences in satisfaction with the neighborhood existed between the two groups, although the MiH participants stated a greater satisfaction than the comparison group. 

The MiH and comparison group interviewees did not differ in their views about their ability to pay monthly housing bills. In both groups, just over half of the participants said that it was “somewhat difficult” or “very difficult” to pay monthly housing bills. The two groups did differ with regard to whether participants reported worrying about being forced to leave their current home, with the comparison group members more likely to report that they worried about being forced out of the home.

The MiH and the comparison group members were similar in health characteristics. No significant differences existed in self-rated health or chronic conditions. A large majority of both groups had access to some health insurance. Both groups had a large proportion of participants reporting a major life event including a death in the family, job loss, divorce, or other events, in the previous year.

## 4. Discussion

This paper provides an overview of the Detroit MiH program and an evaluation aimed at examining whether this program improves housing stability and health over time for the low-income households it serves. Its longitudinal nature will allow us to follow the experiences of a group of homeowners who acquired their homes through the MiH program during the first three years of ownership and assess the threats to their ability to sustain homeownership, including poor health, unexpected life circumstances, loss of income, and opinions about neighborhood and housing conditions. The comparative design of the study will allow us to examine what the MiH homeowners might have experienced if they had not had the opportunity to buy their homes and to analyze the differences in housing stability and health between the MiH participants and a comparison group. The baseline comparisons showed that the two groups were not significantly different regarding socio-demographic characteristics that could place one group at a greater disadvantage. Therefore, the changes in housing stability and health over time and significant differences between the groups during the final interviews can be more confidently attributed to the program. 

Baseline findings highlight the potential challenges to housing stability and health in both groups. Although the MiH homeowners were more satisfied with their housing than the comparison group, nearly half (46.5%) of MiH homeowners felt neutral or somewhat dissatisfied with their housing. We attribute this to the need for major home repairs described by the participants in both groups. MiH homeowners reported worrying less than the comparison group participants about being forced to leave their homes at baseline, indicating that the intervention alleviated the housing precarity caused by tax foreclosure. Still, more than half (55.1%) of the MiH participants reported the ability to pay monthly housing bills as ‘somewhat difficult’ or ‘very difficult,’ even though 81.3% of the MiH participants interviewed had the deed to their homes with no continuing land contract. This may reflect how other housing-related costs including property taxes, repairs and utilities can cause a significant financial burden among low-income households.

### Strengths, Challenges and Limitations

A strength of this research is the longitudinal nature that allows us to examine housing stability and health over time. With a three-year period, we are able to see how households confront challenges over time. Because tax foreclosure takes place after three years of tax delinquency, we will also be able to see whether households are at risk for tax foreclosure again, signaling a pattern that needs to be addressed. In addition, because this study focuses on households where the majority of residents have extremely low incomes, we will observe the sustainability of homeownership for a group that has not been the particular focus of prior research and tends to be housed precariously, usually as cost-burdened renters. Another strength of this study is the partnership with the UCHC, whose positive reputation with clients has helped garner a willingness to participate in our study. Having the UCHC as a partner has provided a front-line perspective that is important for this type of research where programs may adjust or adapt to unforeseen circumstances, such as the COVID-19 pandemic.

Nonetheless, an important challenge for this study has been the recruitment and retention of participants, resulting in a small sample. The literature identifies ‘hard-to-reach’ participants as those who are difficult to involve in research due to circumstances including social and economic distress, low literacy rates, and undocumented immigration status [46,47]. We may find that, as we cannot interview all those we reached at baseline, the interviewees who complete the final interviews represent those with more stable housing and in situations more likely to support housing stability than the MiH or comparison groups on the whole. We will need to make additional efforts to retain interviewees and then to account for the possible bias in our results. Therefore, to try to retain interviewees, we are contacting participants more often than survey procedures usually dictate and ensuring that participants receive compensation for their time. At the conclusion of the study, we will provide participants with the aggregated results and with any additional resources that we have collected over time. Because we may have smaller samples of participants interviewed, we will consider property data as described in the Methods section. Such data will provide us with insight on what happens to the properties in both groups and determine if homeowners experience property loss.

The COVID-19 pandemic has been an additional challenge that has disproportionately affected Black and low-income populations, including most of our participants [48,49]. After the start of the pandemic, we added questions on the effects of COVID-19 on participants’ life events and income, employment, and health outcomes. These will be important factors to consider in our findings as they can undermine homeownership. Furthermore, in response to COVID-19, federal and state governments and state and local courts implemented moratoriums on evictions and tax foreclosures and provided unemployment benefit supplements, direct payments and rent relief. These protections likely helped reduce housing loss for both the MiH and comparison groups. 

A limitation of the study is in the selection of our comparison group of UCHC clients. An ideal comparison group would be made up of a random sample of renters living in houses that went into tax foreclosure in 2017, but we were only able to obtain names and contact information for those renters who had reached the UCHC about the pending tax foreclosures. While the comparison group includes individuals whose homes were foreclosed in 2017, all individuals had sought help through the UCHC to reacquire their homes during the tax auction. Research shows that populations marginalized in the housing market face barriers (e.g., impoverishment, disability, and illiteracy) and are often not aware of or are unable to access aid and resources, such as those offered by the UCHC [50]. Therefore, relying on the UCHC’s client list for the comparison group may not capture the experiences of very low-income renters who are more disconnected from housing assistance programs and who may face greater barriers to housing stability and health after experiencing foreclosure.

## 5. Conclusions

This study is timely as the nation faces a chronic shortage of affordable housing and reduced opportunities for homeownership for low-income groups. The evidence generated can help improve the MiH program by identifying the potential threats to housing instability and the health of homeowners, as well as addressing these issues should the program expand to cover more households at risk of displacement through tax foreclosure. Furthermore, our questionnaires and research approach may help others navigate some of the complexities associated with this type of research.

## Figures and Tables

**Table 1:** Selected characteristics of the MiH participants and comparison group interviewees at baseline interviews.

		MiH (N = 49)	Comparison (N = 39)
*Demographic characteristics*			
Age (mean)		46.6	49.9
	*p* value		0.236
Women (%)		69.4	59.0
Men		30.6	41.0
	*p* value		0.310
African American (%)		93.8	92.1
White		0	5.3
Other		6.1	2.6
	*p* value		0.207
Employed (%)		55.1	46.2
Unemployed		22.5	17.9
Unable to work		20.4	25.6
Retired		2.0	10.3
	*p* value		0.334
*Housing and Housing Instability Characteristics*			
Owner with deed (%)		95.9	33.3
Owner in process		4.1	0
Renter		0	38.5
Other arrangement		0	28.2
	*p* value		0.000
Living in tax foreclosed home (%)		100.0	46.2
Living elsewhere		0	53.9
	*p* value		0.000
Months in current home (mean)		104.7	90.5
	*p* value		0.650
Housing satisfaction (%)			
Very satisfied		6.1	12.8
Somewhat satisfied		46.9	25.6
Neutral		24.5	25.6
Somewhat dissatisfied		20.5	15.4
Very dissatisfied		2.0	20.5
	*p* value		0.024
Neighborhood satisfaction (%)			
Very satisfied		16.3	20.5
Somewhat satisfied		44.9	25.6
Neutral		22.5	28.2
Somewhat dissatisfied		12.2	12.8
Very dissatisfied		4.1	12.8
	*p* value		0.308
Ability to pay monthly housing bills (%)			
Very easy		6.1	5.1
Somewhat easy		18.4	12.8
Neutral		20.4	30.8
Somewhat difficult		40.8	28.2
Very difficult		14.3	23.1
	*p* value		0.510
Worry about being forced out of home (%)			
Strongly agree		6.8	12.8
Agree		9.1	12.8
Neutral		6.8	23.1
Disagree		31.8	48.7
Strong disagree		45.5	0
No answer		0	2.6
	*p* value		0.000
*Health outcomes*			
Good self-rated health (%)		69.4	58.9
Poor self-rated health		30.6	41.1
	*p* value		0.310
No chronic conditions (%)		18.4	12.8
One or more chronic conditions		81.6	87.2
	*p* value		0.480
Has health insurance (%)		97.9	94.9
No health insurance		2.1	5.1
	*p* value		0.428
One or more life event (%)		85.7	87.2
No life event		14.3	12.8
	*p* value		0.842

## Supplementary Materials

The following are available online at https://www.mdpi.com/article/10.3390/ijerph182111230/s1. File S1: Baseline survey for intervention group.
