# Supplementary material for: Threats to and Opportunities for Low-Income Homeownership, Housing Stability, and Health: Protocol for the Detroit 2017 Make-It-Home Evaluation Study
Source: Int J Environ Res Public Health. 2021 Oct 26;18(21):11230. doi: 10.3390/ijerph182111230 (PMC8582874; doi:10.3390/ijerph182111230)
Supplement: Supplementary file 1 [file ijerph-18-11230-s001.zip › ijerph-1410434-supplementary.pdf]

Supplement File S1. Baseline survey for intervention group

A. Housing tenure experience

We will start by asking some questions about your housing in general.

A1. How long have you lived here, in this house?

________ years

A2. Are you still living in the same house that you worked to purchase with UCHC through the Right of Refusal Program?

1. Yes
2. No

[If no, follow up to check whether permanent or temporary:]

Do you expect to return to that house?

1. Yes
2. No

99. Don't know

00. No answer

[if the answer is "don't know," or "no answer," ask the questions that relate to temporarily out after the rest of the A section questions]

[If less than one year in A1, ask A3]

A3. In the past year, how many times have you moved? _______

99. Don't know (do not read out loud)

00. No answer (do not read out loud)

A4. How many years have you lived in this neighborhood? ______ years

A5. How many adults (aged 18-64) currently live in this house (on average 4 days or more per week)? ______ (number)

99. Don't know

00. No answer

A6. How many children currently live in this house (on average 4 days or more per week)? ______ (number)

99. Don't know

00. No answer

A7. How many adults age 65 or older currently live in this house (on average 4 days or more per week)? _____ (number)

99. Don't know

00. No answer

A8. What is your approximate monthly cost for the following housing expenses?

		Don't know	Don't have it	No answer
Land contract/Loan with UCHC	________per month	________	_________	_______
Water	________per month	________	_________	_______
Electricity/gas	________per month	________	_________	_______
Home insurance	________per month	________	_________	_______
Maintenance (snow, lawn, etc.)	________per month	________	_________	_______
Cable	________per month	________	_________	_______
Internet	________per month	________	_________	_______
Other housing expenses	________per month	________	_________	_______

A9. Have you spent money on housing repairs in the past year?

1. Yes
2. No

99. Don't know
00. No answer
[If answer to A9 is no skip to A11]

A10. Approximately how much, did you spent on these housing repair costs this past year?

1. $__________

99. Don't know
00. No answer

A11. How difficult is it for you to meet the monthly payments on your bills related to housing? Is it:

1. Very easy
2. Somewhat easy
3. Neutral (neither easy nor difficult)
4. Somewhat difficult
5. Very difficult

99. Don't know
00. No answer

A12. Do you ever have to borrow money or ask family/friends or others for money to pay for housing?

1. Yes
   [If yes] How much money? _______
2. No

99. Don't know
00. No answer

A13. Since you began in the program to purchase this house, have you fallen behind in paying any of these housing expenses?

	Yes/No	Don't have it	Don't know	No answer
Land Contract with UCHC	___________	________	________	_______
Water	___________	________	________	_______
Electricity/gas	___________	________	________	_______
Home insurance	___________	________	________	_______
Maintenance (snow, lawn, etc.)	___________	________	________	_______
Cable	___________	________	________	_______
Internet	___________	________	________	_______

Other housing expenses? [FILL IN THE TYPE OF EXPENSE AND AMOUNT] ___________

PROBE: Any others? _________________

A14. Since you began in the program to purchase this house, have you been approved for assistance with these housing costs from any the following programs? (read each with its abbreviation and check all that apply)
(If respondent asks what a program is, see sheet that explains each)

1. The Heat and Warmth Fund (THAW) ________
2. Low-Income Self-Sufficient Plan (LSP) ________
3. Water Residential Assistance Program (WRAP) ________
4. State Emergency Relief (SER) ________
5. Other [FILL IN NAME OF PROGRAM] __________________________

99. Don't know
00. No answer

A15. Have you applied for property tax reduction for this house?

1. Yes
2. No

99. Don't know
00. No answer
[If no, skip to question A17]

A16. What type of property tax relief? [do not read the options; let respondent volunteer the answer] PROBE: Anything else?

1. HPTAP (Poverty Tax Exemption)
2. Payment plan
3. Reduction of assessed value
4. Principal Residence Exemption
5. Other _________________

99. Don't know
00. No answer

A17. Is this the first time you have owned a house?

1. Yes
2. No

99. Don't know
00. No answer

[If yes, don't know or no answer, skip to section A19]

A18. How long did you own your last home?
_______ years
99. Don't know
00. No answer

A19. Why did you move? (Do not read this list. Let the respondent volunteer the answer. Check all that the respondent mentions)

1. Sold
2. Transferred house to family or friend
3. Mortgage foreclosure
4. Tax foreclosure
5. Breakup of a relationship or divorce
6. Forming of a new relationship/marriage
7. Major damage to house
8. Moved to Detroit from a different city
9. Other: [FILL IN REASON]_____

99. Don't know
00. No answer

A20. Now I'm going to read a statement and ask how much you agree with it.
I often worry about being forced to move out of my current home. Do you:

1. Strongly Agree
2. Agree
3. Neutral
4. Disagree
5. Strongly Disagree

99. Don't know
00. No answer

A21. I'm going to read another statement and ask how much you agree with it.
I want to stay in this house for the next year. Do you:

1. Strongly Agree
2. Agree

3. Neutral
4. Disagree
5. Strongly Disagree

99. Don’t know
00. No answer

B. Perception of condition of house

Now we are going to ask you a series of questions about the condition of your house.

B1. How satisfied are you with your house? Are you:

1. Very satisfied
2. Satisfied
3. Neutral
4. Dissatisfied
5. Very dissatisfied

99. Don’t know
00. No answer

B2. Thinking about your previous house, how satisfied were you with that house? Were you:

1. Very satisfied
2. Satisfied
3. Neutral
4. Dissatisfied
5. Very dissatisfied

99. Don’t know
00. No answer.

B3. On a scale of 1 to 5 where one is “very satisfied” and five is “very dissatisfied”, how satisfied are you with the following aspects of the interior of your housing.

		1. Very Satisfie	2. Satisfied	3. Neutral	4. Dissatisfied	5. Very Dissatisfied	99. Don't know	00. No answer
a	Natural light							
b	Noise from inside the house [pipes, running water, doors, laundry facilities, etc.]							
c	Amount of space							

d	Safety and security of your house							
e	Problems with insects or animals							
f	Repairs and maintenance needed to the house							
g	Other issues [list if the respondent volunteers these]							

B4. Is the temperature in your house comfortable in the:
winter: (read Yes/No option aloud)

1. Yes
2. No

99. Don't know
00. No answer

B5. Is the temperature in your house comfortable in the:
summer: (read Yes/No option aloud)

1. Yes
2. No

99. Don't know
00. No answer

C. Health Status

Now we are going to ask you some questions about your health and wellbeing.

C1. In general, compared to others your age, would you say your health is:

1. Excellent
2. Very good
3. Good
4. Fair
5. Poor

99. Don't know
00. No answer

C2. Compared to one year ago, how would you rate your health in general now? Would you say your health is:

1. Much better now than one year ago
2. Somewhat better now than one year ago
3. About the same
4. Somewhat worse
5. Much worse

99. Don’t know
00. No answer

C3. During the past 4 weeks, have you had any of the following challenges with daily activities as a result of your physical condition and emotional health?

	Yes	No	Don’t know	No Answer
1. Cut down the amount of time you spent on work or other activities	1	2	99	00
2. Accomplished less than you would like	1	2	99	00
3. Were limited in the kind of work or other activities	1	2	99	00
4. Didn't do work or other activities as carefully as usual	1	2	99	00

C4. During the past 4 weeks, to what extent has your physical or emotional health interfered with your normal social activities with family, friends, neighbors, or groups? Would you say:

1. Not at all
2. Slightly
3. Moderately
4. Quite a bit
5. A lot

99. Don’t know
00. No answer

C5. Has a doctor ever told you that you have [read this list to the respondent and check all that apply]:

a) Chronic bronchitis _____
b) Asthma _____
c) Arthritis or rheumatism _____
d) High blood pressure _____
e) Diabetes _____
f) Heart disease _____
g) Stroke _____
h) Stomach or intestinal ulcers _____
i) Liver problem _____
j) Kidney problem _____
k) Cancer _____
l) Depression _____
m) Sleeping disorders _____
n) Any other long-term condition that has been diagnosed by a health professional:

____

99. Don’t know
00. No answer

C6. Do you have health insurance?

1. Yes
2. No

99. Don’t know
00. No answer

C7. How difficult is it for you to pay health related costs (insurance, prescriptions, out of pocket care, specialty foods, for instance)? Is it:

1. Very easy
2. Somewhat easy
3. Neither easy nor difficult
4. Somewhat difficult
5. Very difficult

99. Don’t know
00. No answer

C8. Do you or any person(s) who normally lives in your household have a physical disability?

1. Yes
2. No

99. Don’t know
00. No answer
If answer is no skip to C10.

C9. Does your house require modifications to help those who normally live in your household cope with their disability?

1. Yes
2. No

99. Don’t know
00. No answer

C10. Have any of the following events happened to you in the past 12 months?

	1. Yes	2. No	99. Don’t know	00. No answer
a. Did you lose, quit or change your job?				
b. [If have children] Did you have a child, or adopt a child?				
c. Did you have serious financial difficulties or worries?				
d. Did you have any legal difficulties or problems with the police?				

e. Did you divorce or separate from your spouse or partner?				
f. Did you have a serious illness or accident?				
g. Has your husband, wife, or partner died? [modify to fit what the respondent has already told you]				
h. Has anyone else close to you died?				
I. Have you experienced any other major events in the past 12 months? (if yes) What were these? PROBE: Anything else?<br>_____________<br>_____________<br>_____________				

C11. Thinking about the past year and the amount of stress in your life, would you say that most days are…

1. Not at all stressful
2. Not very stressful
3. Neutral
4. Fairly stressful
5. Very stressful

99. Don't know
00. No answer

D. Neighborhood conditions

Now we are going to ask you some questions related to the neighborhood you live in.

D1. How satisfied are you with the neighborhood where you live? Are you:

1. Very satisfied
2. Somewhat satisfied
3. Neutral
4. Somewhat dissatisfied
5. Very dissatisfied

99. No answer
00. Don't know

D2. Thinking about the last neighborhood you lived in, how satisfied were you with that neighborhood? Would you say you are:

1. Very satisfied
2. Somewhat satisfied
3. Neutral
4. Somewhat dissatisfied

5. Very dissatisfied

99. No answer

00. Don’t know

D3. On a scale of 1 to 5 indicate how much do you strongly agree--1-- or strongly disagree--5-- with the following statements:

	Neighborhood status	Strongly agree (1)	Agree (2)	Neutral (3)	Disagree (4)	Strongly disagree (5)	Dont know (99)	No answer (00)
a	The neighborhood where I live is child friendly							
b	The neighborhood where I live is senior friendly							
c	Violence and crime are big issues in my neighborhood							
d	I feel safe when I go home at night in my neighborhood							
e	Blight and abandoned buildings are a big issue in my neighborhood							

D4. Now I am going to ask how satisfied you are with the different aspects of your neighborhood on a scale of 1 to 5. Would you say you are very satisfied--1-- or very dissatisfied--5-- with the following:

	Neighborhood Aspect	Very Satisfied (1)	Satisfied (2)	Neutral (3)	Dissatisfied (4)	Very Dissatisfied (5)	Dont know (99)	No answer (00)
a.	Parks and green space							
b.	Amount of traffic							
c.	Police protection							
d.	Personal safety							
e	Access to a commercial district							

	with stores and services.							
f	Provision of public services (e.g. lighting, street repair, trash collection)							

D5. Now I am going to read some statements that people might make about their neighborhood. Please tell me on a scale of 1 to 5 if you would strongly agree--1--or strongly disagree—5:

		Very Satisfied (1)	Satisfied (2)	Neutral (3)	Dissatisfied (4)	Very Dissatisfied	Dont know (99)	No answer (00)
a	I think my neighborhood is a good place for me to live.							
b	My neighbors and I want the same things from the neighborhood							
c	I can recognize most of the people who live on my block							
d	Very few of my neighbors know me							
e	I care about what my neighbors think of my actions							
f	If there is a problem in this neighborhood, people who live here can get it solved							
g	It is very important to me to live in this particular neighborhood							
h	People in this neighborhood generally don’t get along with each other							

E. Experience with the program

We’d like to learn more about your experience purchasing your home out of tax foreclosure with the United Community Housing Coalition (“UCHC”).

Think back to your experience acquiring your deed or entering into a land-contract with UCHC.

E1. What worked well? _______________

PROBE: What else? Anything else?

____

____

____

E2. What didn't work well? ____________________

PROBE: What else? Anything else?

____

____

____

E3. Think back now about your transition to homeowner.

What is going well? __________________

PROBE: What else? Anything else?

____

____

____

E4. What is not going well? _________________

PROBE: What else? Anything else?

____

____

____

E5. What feedback would you have for the UCHC staff?_______________

PROBE: What else? Anything else?

____

____

____

E6. Is there anything else you'd like to share with us about your experience acquiring the deed or entering into a land contract with UCHC or about your transition to becoming a homeowner?

PROBE: Anything else?

____

____

____

F. Household Information

Now I'm going to ask you some questions about your household and yourself.

F1. In the past 12 months, did you, yourself, receive income from any of the following (check all that apply):

1. Wages (hourly pay, salary)
2. Unemployment compensation
3. Pension
4. Social Security (retirement)

5. Social Security Disability
6. Supplemental Security Income
7. VA benefits
8. Family Independence Agency (FIA) (i.e. DHS)
9. Bridge card
10. Child support/alimony
11. Rental income
12. Help from family/friends
13. Workers compensation
14. Other

99. Don’t know
00. No answer

F2. Based on the sources of income you just identified, how much income did you receive last month? ____________

99. Don’t know
00. No answer

F3. Does anybody else in the household receive income? This includes any of the sources we mentioned.

1. Yes
2. No

99. Don’t know
00. No answer
[If no skip to F5]

F4. [IF YES] How much income did each household member receive this past month?
Household Member 1 __________
Household Member 2 __________
Household Member 3 __________
Household Member 4 __________
Household Member 5 __________
Household Member 6 __________
Household Member 7 __________
Household Member 8 __________
Household Member 9 __________
Household Member 10 __________
99. Don’t know
00. No answer

F5. Did you file income taxes for 2018?

1. Yes

2. No

99. Don’t know

00. No answer

[If participant says no, skip to F7]

F6. If you filed income taxes for 2018 did you receive tax deductions, exemptions or credits from the following sources? (if respondent asks what one of these are, see sheet that explains each)

1. Homestead Property Tax Credit (State) ________
2. Home Heating Credit (State) ________
3. Other [FILL IN NAME OF TAX RELIEF] ______________________________

99. Don’t know

00. No answer

F7. How satisfied are you with (you/your household’s) present financial situation? Are you:

1. Very satisfied
2. Satisfied
3. Neutral
4. Dissatisfied
5. Very dissatisfied

99. Don’t know

00. No answer

G. Respondent Information

G1. How old are you? ______

99. Don’t know

00. No answer

G2. What is your current marital status? (check what the respondent says; do not read list)

1. Single
2. Married or living together
3. Divorced or separated
4. Widowed
5. Other: _________

99. Don’t know

00. No answer

G3. What is your highest educational attainment? (check what the respondent says; do not read list)

1. Less than a high school diploma
2. High school degree or equivalent (e.g. GED)
3. Some college, no degree

4. Associate degree (e.g. AA, AS)
5. Bachelor's degree (e.g. BA, BS)
6. Graduate Degree (e.g. Masters, PhD, professional degree)

99. Don't know
00. No answer

G4. What is your employment status? (check what the respondent says; do not read list)
(if respondent says "employed", ask "Full-time or part-time?"; if the respondent says "unemployed", ask "are you currently looking for work?")

1. Employed full time (40 or more hours per week)
2. Employed part time (up to 39 hours per week)
3. Unemployed and currently looking for work
4. Unemployed and not currently looking for work
5. Student
6. Retired
7. Homemaker
8. Self-employed
9. Unable to work
10. Other: _________

99. Don't know
00. No answer
[IF DID NOT ANSWER 1 OR 2 IN PREVIOUS QUESTION SKIP TO G6]

G5. What is your current employment condition? Are you a:

1. Permanent employee
2. Temporary employee
3. Contract employee
4. Other ____________

99. Don't know
00. No answer
G6. What is your gender?

1. Female
2. Male

99. Don't know
00. No answer

G7. What is your race/ethnicity?

1. African American
2. White
3. Latino/a or Hispanic

4. Asian
5. Biracial
6. Other

99. Don’t know
00. No answer

[END OF SURVEY]
